# The Treatment of Whooping-Cough with Quinine

**Published:** 1873-04

**Authors:** B. F. Dawson

**Affiliations:** Clinical Lecturer on the Diseases of Children in the Medical Department of the University of New York; Physician for Children to the Demilt Dispensary; to the New York Free Dispensary for Sick Children, etc.


*The Treatment of Whooping-Cough with Quinine.* By B. F. DAWSON, M.D., Clinical Lecturer on the Diseases of Children in the Medical Department of the University of New York; Physician for Children to the Demilt Dispensary; to the New York Free Dispensary for Sick Children, etc.

I am well aware that every therapeutical assertion, especially concerning pertussis, is to be accepted with the utmost caution, and that value can be placed upon such only as have been well tried and are based upon careful clinical study.

I deem it, however, the duty of every physician, after having carefully observed the value of any one therapeutical agent in the cure of some one disease, to make the same known to the profession, whereby its real value may be proven or its worthlessness exposed.

In advocating the use of quinine in pertussis, I am fortunate in being able to support my own experience with that of one whose name is well known on both continents, and whose contributions to the progress of medical science are always received as the teachings of one speaking with authority. I refer to Professor Binz, of the University of Bonn, Germany.

In 1870, a paper on "The Use of Quinine in the Diseases of Children" was contributed by him to this journal, (Vol. III, No. 1, May, 1870,) in which he advocated the use of quinine in pertussis, and stated that in his hands it had accomplished valuable results. Considering pertussis to be a neurosis of the pneumogastric nerve, caused by infectious and irritating mucus that has accumulated in the larynx and pharynx, and having found by experiments that quinine destroyed, even when highly diluted, all structures found in normal mucus, he supposed, without taking into consideration the more intimate morphological connection, that the mucus of pertussis also would be affected in a similar manner by quinine.

In this he was not disappointed, the trial equaling his expectations.

In the clinic for children's diseases which he held in Bonn, he says: "I have treated for the past two years all the cases of pertussis, without any exception, with quinine. The best proof of its good effects is seen in the fact that those in charge of the little patients repeatedly call again for the 'bitter medicine' whenever they have succeeded, either by coaxing or force, in administering it to them. There was a most striking difference to be seen in those whom it was impossible by any means to induce to swallow the solution of quinine. In these cases the whooping-cough assumed its regular obstinate course; in the others, although living in all other respects under perfectly similar circumstances, the paroxysms were always reduced in frequency and severity."* These good results with quinine, he stated, could only be obtained by strictly observing the following conditions: "*It should be given in solution; the dose should not be too small, and should not be administered in a vehicle that will prevent it from coming in contact with the mucous membrane in its passage through the pharynx;*" and the neglect of one or all of these rules he considers the reason why other observers have seen no positive results from the use of this drug. Certainly it is not just to condemn a remedy as ineffectual when it is not employed in the proper manner.

The assumption that pertussis is a specific local catarrh, caused by a fixed contagion admitted from without, Prof. Binz thinks, admits of being hypothetically explained by the fact of adults being almost unexceptionally exempt from it. "The stronger development of the epithelium may be regarded as a protection against the affection of the mucous membrane. This greater development in children probably takes place quicker if those parts of the throat, from pertussis, have been in a hyperæmic condition for weeks, and thus it is easy to comprehend how the immunity originates after the affection has once been surmounted."†

Still another cause of pertussis has been advanced. Dr. Letzerich, of Germany,‡ in 1871, asserted in a paper on the subject that he had discovered a form of fungoid growth which vegetates in the epithelium of the air passages, and by its irritation causes the convulsive attacks of coughing. The expectorated mucus in patients suffering from pertussis, he says, contains masses of brownish-

* American Journal of Obstetrics and Diseases of Women and Children, vol. iii, No. 1, page 8.

† Loc. cit., pages 9, 10, foot notes.

‡ Quarterly Journal Medical Science and American Journal of Obstetrics, vol. iv, page 761.

red spores, with occasional threads of mycelium, which in the latter stages of the disease becomes very abundant. These observations were made by experiments upon rabbits into whose tracheæ he introduced the fungus; in a short time the latter became affected with a noisy and violent cough—in fact, a genuine whooping-cough. The rabbits thus affected were killed and examined, and their air passages were found to contain the same fungus as that found in the sputa of human subjects; in fact, the mucus presented precisely the same appearance.

This fungus theory is certainly a very plausible and possible one, and one that even seems to be proven by the effects of therapeutical measures directed against the development of the fungus.

The fact that narcotic remedies do sometimes greatly influence whooping-cough, does not, however, weaken this latter theory, for by their use the sensitiveness of the pneumogastric nerve, and of the whole nervous system, is so benumbed as to but feebly appreciate or respond to the irritation in the pharynx and larynx.

Quinine, it is well known, has a powerfully destructive effect on true fungi and fungus germs—hence its great power over septic or zymotic affections; and why should it not influence the growth of the fungus of pertussis?

This theory of Dr. Letzerich tends to strengthen our belief in the appropriateness of Prof. Binz's treatment, for if the fungus theory is the correct one, then quinine with its destructive effects on fungoid matter may certainly be considered a most appropriate remedy.

Another advocate of the use of quinine in pertussis, a short time after Prof. Binz's views had been made public, was Dr. Breidenbach, who published a paper (noticed in *The Practitioner*, Feb. 1871, London,) on the efficacy of the hydrochlorate of quinine in a violent epidemic of 1870. In all pure cases he states its effects were really surprising, as soon as he had from precise observations determined the proper dose and mode of administration, in which latter point he thinks lies a great part of his success. The amount administered by him—the age of the subjects varying from three weeks to eight years, and the violence of the attacks being very different in different cases—varied from 1½ to 15½ grains. No other remedy than quinine was employed, and some of the children were freely exposed by poverty to the injurious effects of the weather. In the worst cases, he says, after the use of the remedy for forty-eight hours, the frequency and violence of the attacks diminished.

With such strong testimony in favor of the quinine treatment of pertussis, it is somewhat surprising that nothing, or very little, has been done in this country to test its value. Even in our text-books on diseases of children, nothing is said in reference to the use of quinine in whooping-cough, and in such recent works as the last editions of Lewis Smith's and Meigs and Pepper's books, the omission still continues, notwithstanding that the articles already referred to appeared in 1870–1 in an American journal, the only one devoted to diseases of children published in the English language. We can but trust that in the future editions the subject will receive proper attention.

Having opportunities for testing the value of anything new in infantile therapeutics, I determined after having read Prof. Binz's paper, to apply his treatment in all cases of whooping-cough coming under my care, at the two dispensaries with which I am connected, as well as in my private practice.

I did not have long to wait. On December 4th, 1871, the first case came to my class at the "Free Dispensary for Sick Children;" the following is the record of it, and five of the most striking cases.

CASE I. Annie C——, 4 years. First whooped three nights ago; since then five or six times a day; is worse at night, paroxysms very soon ending in vomiting. Ordered solution of the sulphate of quinine of fifteen grains to the ounce of water, a tea-spoonful to be given every two hours. No other treatment. To return on the 6th.

*Dec.* 6.—Mother states that she vomited the first dose, which was given at 1 P. M., and considerable thick phlegm. Had no whoop until just before giving the evening dose at 7; also once at night. The paroxysms were not so severe. She whooped once at 9 this A. M., but much softer, without any nausea. Ordered half a tea-spoonful of the quinine solution in one of water every two hours, and to return on the 8th.

*Dec.* 8.—Child greatly improved in appearance. Mother states that she has whooped but once since the 6th, and that was on the same evening. Ordered to continue medicine, and return on the 10th.

*Dec.* 10. Has not whooped since 6th. Ordered to continue the quinine in same manner, but only three times daily for one week. To return if she whoops again. This she did not do; so she was registered as cured.

CASE II. Margaret M——, 7 years. Brought to the same institution Dec. 18, 1871. First whooped five nights ago, (Dec. 11); since then has grown worse, and now whoops almost every hour. Had an attack while in the dispensary, which was very severe, and was followed by vomiting. Ordered solution of the sulphate of quinine, ten grains to the ounce of water, a teaspoonful every two hours daily. To return Dec. 20.

*Dec.* 20. Vomited first and second doses, with it considerable stringy sputa, more than in previous attacks; a slight whoop occurring each time. Since then has whooped but twice during yesterday, once during the night, and on rising this A. M. Attacks not so severe. Medicine ordered to be continued.

*Dec.* 24. Child has whooped but once daily in the evening since 20th. Continue treatment.

*Dec.* 28. Has not whooped for two days. Continue treatment for one week.

*Jan.* 5. No return of whoop. Discharged cured.

CASE III. Bernard W——, 22 months. Healthy child. Brought to the Demilt Dispensary Dec. 20, 1871. Whooped first on the previous evening, since then two or three times. Ordered quinine in solution, five grains to the ounce, a teaspoonful every hour.

*Dec.* 24. Child whooped but twice since taking the quinine on the same night, Dec. 20, and vomited the first three doses; with them considerable tough sputa. Continue treatment.

*Dec.* 28. No return of the whooping since the night of the 20th. Discharged cured.

CASE IV. Albert F——, 10 years. Was brought to my office by his father, Jan. 3, 1872; he having whooped twice during the preceding night. Ordered quinine sulphate, ten grains to the ounce, a teaspoonful every hour. To call in two days.

*Jan.* 6. Whooped once very slightly in the night of the 3d. Not once since. First dose nauseated; coughed up considerable thick phlegm after first few doses. Ordered to continue the quinine for one week. No whooping occurred during that period.

CASE V. George F——, 4 years. Brought to the Demilt Dispensary Jan. 11, 1872; having had the whooping-cough for the past two weeks. Paroxysms occur several times daily, and so frequent at night as to keep all awake. Vomits frequently, and shows markedly the effects of the disease. Ordered solution quinine, ten grains to the ounce, a teaspoonful everyahour during the day, and at night when awake.

*Jan.* 13. Much improved, the paroxysms not lasting so long or being so severe; ending at first in coughing up thick phlegm, but not so much now. Had three attacks during the night. Continue treatment.

*Jan.* 17. Has greatly improved; has not whooped for two days until this morning, when his mother thought he did. Ordered to continue the quinine.

*Jan.* 23. Has not whooped since last visit. Is "wonderfully well," as the mother expressed it. Was not again seen.

Case VI. George W——, 3 years. Came under my care Feb. 24, 1872. Had two whooping attacks during preceding night, and once on the morning of visit. Is in good health otherwise. Ordered quinine, ten grains to the ounce of water, a teaspoonful every hour.

*Feb.* 26. Whooped twice on the 24th, and once yesterday noon, though not so severely, and easily coughed up thick mucus. Last night had but one severe attack of coughing, but did not whoop.

*Feb.* 28. Whooped once very slightly night before last; none since.

*Feb.* 30. Has not whooped since last visit.

*March* 3. Has not whooped since the 28th. Ordered to discontinue the quinine, and to be brought to me should the whooping return. The child was not again seen, and I subsequently learned that the whooping did not return.

The above six cases have been selected out of sixteen cases of pertussis seen by me during the past year, in which quinine was the only remedy used; the remaining ten presenting similar histories. Out of the sixteen cases, the shortest cure was effected in one day, and the longest in twenty days. In but two cases have I been disappointed in the efficacy of the quinine. They were two dispensary cases; and from the fact that one, a little girl, was under the care of her father, and the other was a "farmed-out" infant of twelve months, I am inclined to attribute the failure to the negligence of those in charge of them, the quinine not being given to them as frequently as ordered. In both these cases, however, there was some palliation of the paroxysms.

In regard to the administration of so disagreeable a remedy, I found that, though frequently there was some difficulty in getting the children to take it, yet it was exceptional for them to resist after the first two or three doses, and in only a very few did it cause vomiting. The direction to give the children a piece of an orange or a little sugar five or six minutes after taking the quinine, doubtless, had considerable to do with their seeming willingness to take the "bitter medicine."

As to how the quinine so very remarkably influences this most troublesome and severe disease several theories might be advanced. If the fungus theory of Dr. Letzerich be the correct explanation of pertussis, then we can readily account for its efficacy, in the already mentioned fact of its destructive influence on fungoid development, and consequently its power consists in removing the cause of local irritation, which gives rise to the reflex phenomena evidenced in the whooping.

The above theory and explanation carries with it considerable weight, and, it appears to me, should be accepted until disproved, or a more convincing pathological explanation of pertussis is given.

For my own part, I accept it, and in consequence consider pertussis an affection of the mucous membrane of the pharynx and larynx, and the "whooping" as simply reflex. And the fact that almost all remedies given for other than their local effects, have either signally failed or but partially succeeded, strengthens this hypothesis.

Nevertheless, I do not attribute the rapid cure effected by quinine to the simple destruction of the fungus, but also to its nauseating bitter taste. In every case of pertussis, it will be conceded by all, there is an abnormal secretion of a thick tenacious mucus from the mucous membrane of the pharynx, (whether this secretion is due to simple catarrhal or reflex hyperæmia, or to fungoid development, it matters not,) which may or may not excite a paroxysm of whooping, but which certainly aggravates and prolongs the latter, as may be proved by the fact that the paroxysms invariably cease the moment this mucus is removed either by the coughing, vomiting, or the finger. Now, the effect of a small amount of a solution of quinine, when taken into the mouth and swallowed, is instantly, from its bitter and nauseating taste, to excite a free secretion of thin mucus from the buccal mucous membrane and the salivary glands, and this softens and renders easy of dislodgment the tenacious mucus referred to. The frequent repetition of the quinine, therefore, keeps up this free secretion, and thus prevents the mucus from becoming tenacious and difficult of dislodgment. At each act of coughing, therefore, the accumulated mucus is readily loosened and expectorated, and unobstructed inspiration obtained. The rapid loosening of the cough, the briefness of the attacks in comparison with those previous to the administration of the quinine, and the easy expectoration, certainly tend to favor the correctness of the above theory.

The failure of quinine against pertussis, in the hands of others who have tried it, is undoubtedly to be attributed to the manner of its administration—either in large doses at long intervals, or in the form of pills; in either case, therefore, the local effects upon which I place the greatest value are not obtained. While writing this paper, a friend, whose practice is largely amongst children, informed me that he met with no success with quinine in pertussis; but on his informing me that he had always given it in large doses, morning and evening, I attributed his failure to that fact.

The object with which I have written this paper is to call the attention of the profession to this treatment of pertussis, and invite them to give it a careful trial, feeling convinced that if the following rules are carefully observed, few, if any, will be disappointed in their results.

1. Give the quinine (sulphate or hydrochlorate) dissolved by acid in pure water only. For children under 3 years, from gr. v. to gr. viij., and for older children and adults, from gr. x. to gr. xij. to the ounce.

2. Give not less than a teaspoonful *every single*, or, at the longest, every two hours during the day, and whenever cough comes on in the night.

3. Give nothing afterward for some minutes to destroy the taste or to wash out the mouth.

4. Continue giving it notwithstanding the first doses may be vomited.

5. Be sure that the quinine is pure and thoroughly dissolved.

*Appendix:*—Since the above paper went to press, two cases of whooping have come under my care. One, a boy of three years, who was brought to my clinic in the University on Feb. 8; had whooped severely four and five times daily, and as often during the night. He had an attack in the presence of the students in the lecture-room. He was ordered quinine gr. v. to the ounce of water, a teaspoonful every hour daily. No other treatment. He was again brought to the clinic the following Saturday, Feb. 15, when his father stated that the attacks at once grew less severe and frequent after taking the quinine, and that on Wednesday day and Thursday (fifth and sixth days) he whooped but once in each twenty-four hours, very slightly. The medicine gave out on Thursday evening, however, and since then the whooping has increased in severity and frequency. The other case was a little girl two years old, who was brought to the Free Dispensary for Sick Children on Feb. 11. She had whooped for three days, five or six times during the day and night. Was ordered quinine as in the preceding case. Was again seen Feb. 15, at my clinic, and shown to the class. In this case the mother confessed to having been negligent in giving the medicine, not having given it oftener than four or five times during the day; and yet she said the child had greatly improved, and had whooped but once or twice during the night time only since taking the quinine. Both of these cases were also seen previous to and after treatment by Dr. P. B. Porter and Dr. Beverly Robinson, and being the last cases under my care, are a valuable addition to the preceding report of the six out of my first cases.

In the foregoing paper I wish to be understood as advocating the value of quinine in curing the "whooping" chiefly, the cough in many of the cases lasting for some time after the whooping ceased, and which requires the usual treatment for bronchial catarrh.—*American Journal of Obstetrics and Diseases of Women and Children, Feb.*, 1873.

